# Prevalence, Clinical Features, and Outcome of *Pseudomonas* Bacteremia in Under-Five Diarrheal Children in Bangladesh

**DOI:** 10.1155/2014/469758

**Published:** 2014-03-09

**Authors:** Farhana Akram, Mark A. C. Pietroni, Pradip Kumar Bardhan, Samira Bibi, Mohammod Jobayer Chisti

**Affiliations:** ^1^Pharmacy Department, East West University, Dhaka, Bangladesh; ^2^Public Health for South Gloucestershire, South Gloucestershire, Badminton Road, Yate, BS37 5AF, UK; ^3^Dhaka Hospital, International Centre for Diarrhoeal Disease Research, Bangladesh (icddr,b), 68 Shaheed Tajuddin Ahmed Sarani, Mohakhali, Dhaka 1212, Bangladesh; ^4^Centre for Nutrition and Food Security (CNFS), International Centre for Diarrhoeal Disease Research, Bangladesh (icddr,b), Dhaka, Bangladesh

## Abstract

We sought to evaluate the prevalence, associated factors, and outcome of under-five diarrheal children with either sex having *Pseudomonas* bacteremia. A retrospective chart review of under-five diarrheal children admitted to the Dhaka Hospital of the International Centre for Diarrhoeal Disease Research, Bangladesh (icddr,b), from January 2011 to December 2011 was performed using an online hospital management system. Children with *Pseudomonas* bacteremia constituted the cases (*n* = 31), and the controls (*n* = 124), without *Pseudomonas* bacteremia, were randomly selected. The prevalence of *Pseudomonas* bacteremia was 1% (31/5,179). The *Pseudomonas* was multidrug resistant but was 84% sensitive to ceftazidime and 100% to imipenem. The case-fatality rate was significantly higher among the cases than the controls (26% versus 5%; *P* = 0.003). In logistic regression analysis, after adjusting for potential confounders such as severe wasting, severe underweight, severe pneumonia, and young age (11.71 (4.0, 18.0) months), the cases more often presented with absent peripheral pulses in absence of dehydration (95% CI = 2.31–24.45) on admission. This finding underscores the importance of early identification of this simple clinical sign to ensure prompt management including fluid resuscitation and broad spectrum antibiotics to help reduce morbidity and mortality in such children, especially in resource-poor settings.

## 1. Introduction

Sepsis remains a leading cause of morbidity as well as mortality in the pediatric population [1–5]. Most of these deaths occur in developing countries [6–8].* Pseudomonas*, a facultative anaerobe Gram-negative organism that is commonly discovered in soil, water, and plants, seldom causes illness in healthy people. However,* Pseudomonas* sepsis often occurs in patients with burns, malignancy, or immunodeficiency or in preterm infants. Most of these infections are nosocomially acquired [9, 10].* Pseudomonas* infection is clinically indistinguishable from other forms of Gram-negative bacterial infection. For this reason, patients with* Pseudomonas* infection often receive empirical antibiotics that are not sufficiently active against* Pseudomonas*, especially before culture results and antibiotic sensitivities become available [11–13]. Despite recent improvements in therapy,* Pseudomonas* bacteremia remains fatal in more than 20% of cases [14]. In a recent large multicentre study of all age groups,* Pseudomonas* bloodstream infection (BSI) was multidrug resistant (MDR) and associated with crude mortality rates of 39% in all patients and 48% in intensive care unit patients [15]. Mortality is even higher when children with* Pseudomonas* bacteremia also have diarrhea [16]. Thus, children with* Pseudomonas* bacteremia and diarrhea need careful attention. An understanding of the factors associated with* Pseudomonas* bacteremia in diarrheal children may help clinicians in identifying and managing such children promptly and efficiently in order to reduce morbidity and mortality especially in resource-poor settings. However, there is little data on factors which may predict* Pseudomonas* bacteremia in children with diarrhea. The aim of our study was to identify the prevalence, clinical predictors, and outcomes of* Pseudomonas* sepsis in under-five diarrheal children.

## 2. Materials and Methods

### 2.1. Ethics Statement

Our research did not involve any interviews with patients or caregivers and it was solely a chart analysis. The data were anonymised before being received by us.

### 2.2. Type of Study

This study involved a retrospective analysis of data extracted from the hospital management system (SHEBA), an online data base of the Dhaka Hospital of icddr,b.

### 2.3. Study Population and Site

We conducted our study at the International Centre for Diarrhoeal Disease Research, Bangladesh (icddr,b). Every year it deals with around 140,000 patients; most of the patients have diarrhea with or without other complications [17]. After arrival at hospital triage nurses obtain the patient's medical history and make a rapid assessment, focusing on the severity of disease as well as other health complications. Within half an hour of admission, all patients are evaluated by duty physicians and receive appropriate treatment. Patients with complications of diarrhea or those with respiratory distress, cyanosis, apnea, hypothermia, sepsis shock, impaired consciousness, convulsion, severe pneumonia with hypoxemia, or respiratory failure are admitted to the Intensive Care Unit (ICU) or Longer Stay Ward (LSW) of the Dhaka Hospital of icddr,b. The majority of the patients come from a poor socioeconomic background. In addition to clinical services, the hospital also conducts research on various infectious diseases such as diarrhea, pneumonia, and sepsis and malnutrition.

### 2.4. Study Design

For this study, we used an unmatched case-control design. Data on diarrheal children of both sexes, aged 0–59 months, admitted to ICU or LSW of icddr,b from January 2011 to December 2011, who had a blood culture, were extracted from SHEBA. Under-five diarrheal children with* Pseudomonas* bacteremia constituted the cases, and those without* Pseudomonas* bacteremia constituted controls. Controls were randomly selected from the children without* Pseudomonas* bacteremia by computer randomization using SPSS (version 17.0; SPSS Inc., Chicago) from the personal computerized data source of this study. A 1 : 3 unmatched case-control ratio was used to increase the statistical power of our analyses.

### 2.5. Measurements

Case report forms (CRF) were developed, pretested, and finalized for data acquisition. Characteristics analyzed include demographics (age, gender, and place of residence), fever, cough, history of difficulty breathing, acute watery diarrhea, pneumonia, absent peripheral pulses even after correction of dehydration or in absence of dehydration, hospital acquired infection (any new infection documented after 48 hours of admission), severe wasting, severe underweight, severe stunting, abnormal mental status (disorientation or lethargy or convulsion), radial pulse, respiratory rate, temperature and outcome.

### 2.6. Analysis

All the data were entered into SPSS for Windows (version 17.0; SPSS Inc., Chicago) and Epi-Info (version 6.0, USD, Stone Mountain, GA). Differences in proportions were compared by the chi-square test. In normally distributed data, differences of mean were compared by Student's *t*-test; the Mann-Whitney test was used for comparison of data that were not normally distributed. A probability of less than 0.05 was considered statistically significant. Strength of association was determined by calculating the odds ratio (OR) and 95% confidence intervals (CIs). In identifying predictors of* Pseudomonas* sepsis in under-five diarrheal children with pneumonia, variables were initially analyzed in a univariate model, and then independent predictors were identified using logistic regression after controlling for covariates.

## 3. Results

**Figure 1:**
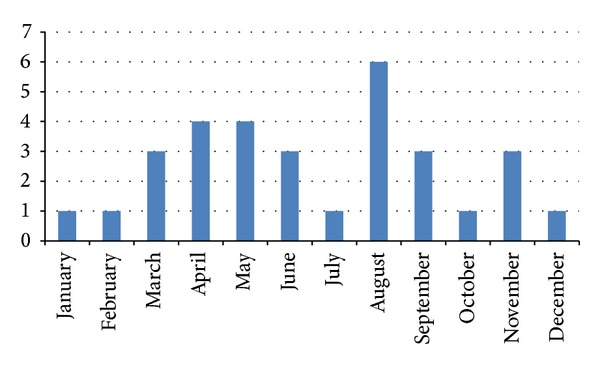
Monthly distribution of* Pseudomonas* bacteremia (number) in 2011 in Dhaka Hospital.

Among a total of 5,179 under-five diarrheal children who were available for the evaluation, 31 met the case definition (1%) and 124 controls were randomly selected from the rest. The case fatality was significantly higher among the cases than the controls (Table 1). The* Pseudomonas* was multidrug resistant (Table 3) but was sensitive to imipenem (100%) and ceftazidime (84%). In a logistic regression analysis, after adjusting for potential confounders (such as young age (11.71 (4.0, 18.0) months), severe pneumonia, severe underweight, and severe wasting), an absent peripheral pulse in the absence of dehydration has been revealed as an independent predictor of* Pseudomonas* bacteremia in diarrheal children (Table 2). The distributions of sex, difficulty in breathing, cough, respiratory rate, abnormal status, and hospital acquired infection among the cases and the controls were comparable (Table 1). There was no bacteremia among the controls that had absent or uncountable peripheral pulses. The monthly distribution of* Pseudomonas* bacteremia has been shown in Figure 1.

## 4. Discussion

Our observation of a higher case-fatality rate in under-five diarrheal children with* Pseudomonas* bacteremia is an important finding, although it is not surprising. Treatment of children with* Pseudomonas* bacteremia often requires prompt adherence to appropriate parental broad spectrum antibiotics. The first line of management of sepsis in our hospital is the combination of parental ampicillin and gentamicin, and the second line is the combination of ceftriaxone and gentamicin. These combinations are used in our hospital as previous studies in Bangladesh have revealed that these combinations are very effective in most children with clinical sepsis [18]. Third and fourth lines of treatment of sepsis in our hospital are the combination of ceftazidime and amikacin or imipenem alone, respectively. In cases of hospital acquired infection, ceftazidime and amikacin together and imipenem are the first and second lines of treatment. Thus, apart from 1 patient with hospital acquired* Pseudomonas* bacteremia, no other patients with* Pseudomonas* bacteremia in our series received appropriate antibiotics before the availability of the blood culture report. The culture reports are usually received 72 hours after admission in our hospital. The resulting delay in the use of appropriate antibiotics may contribute to the higher mortality in diarrheal children with* Pseudomonas* bacteremia. A number of studies have revealed that sensitive but delayed antimicrobial therapy in children with* P. aeruginosa* bacteremia is often associated with septic shock resulting in higher mortality [10, 19] and underscores the importance of appropriate initial antimicrobial treatment [20, 21].

Our observation of absent or uncountable peripheral pulses in absence of dehydration or after correction of dehydration, as an independent predictor of* Pseudomonas* bacteremia in under-five diarrheal children, is also understandable. Absent peripheral pulses, after appropriate correction of dehydration and maintenance of hydration, are likely to be due to compromised peripheral circulation secondary to* Pseudomonas* sepsis-related phenomena, such as impaired vasoregulation and cardiac dysfunction. This finding is consistent with our earlier studies [22]. Thus, our findings suggest that under-five diarrheal children with absent or uncountable peripheral pulses in the absence of dehydration or after full correction of dehydration should be treated aggressively with an antipseudomonal parental cephalosporin (ceftazidime) or with carbapenem (imipenem) in addition to routine rapid fluid resuscitation.

We failed to observe any significant difference in gender, fever, cough, history of difficulty in breathing, hospital acquired infection, abnormal mental status, radial pulse, and respiratory rate among the groups. This might be due to the small sample. However, failure to identify any statistical difference in hospital acquired infection among the groups might be due to the fact that there was a lack of documentation of hospital acquired infection in the digital record system (SHEBA). Our study has potentially two important limitations. First, the retrospective nature of the study in identifying* Pseudomonas* bacteremia prevented us from collecting information on a broader range of variables that may have been potential additional predictors. Second, the limitation in the number of patients included in this study might have reduced the ability to identify more subtle differences between the groups and identify further relevant predictors of* Pseudomonas* bacteremia.

In conclusion, the results of our data analyses suggest that the prevalence of* Pseudomonas* bacteremia is low but is associated with a high case-fatality rate in under-five diarrheal children. Absent peripheral pulse in the absence of dehydration or after full correction of dehydration was the best predictor of* Pseudomonas* bacteremia in under-five diarrheal children. Identification of this simple clinical predictor of* Pseudomonas* bacteremia may help prompt as well as aggressive management with rapid fluid infusion and broad spectrum antibiotics in diarrheal children and result in reduced morbidity and mortality especially in resource-poor settings. However, a further study with a larger sample and a prospective design is imperative to confirm our observation.

## Figures and Tables

**Table 1:** Admission characteristics of diarrheal children with (cases) and without* Pseudomonas *bacteremia (controls).

Variables	Cases *n* = 31	Controls *n* = 93	Unadjusted odds ratio (95% Cl)	*P*
Age (median, IQR)	11.71 (4.0, 18.0)	8.93 (5.03, 19.0)	—	0.354
Respiratory rate/minute (mean ± SD)	47.45 ± 14.84	46.73 ± 12.78	0.720 (−5.48–0.92)	0.806
Gender (male)	20 (65)	59 (63)	1.05 (0.42–2.73)	0.914
History of the cough	10 (32)	33 (36)	0.87 (0.33–2.20)	0.913
History of difficulty breathing	8 (26)	21 (23)	1.19 (0.40–3.29)	0.903
Presence of fever	12 (39)	38 (41)	0.91 (0.36–2.26)	0.999
Absent peripheral pulses even after correction of dehydration or in absence of dehydration	10 (32)	6 (7)	6.91 (1.97–25.41)	<0.001
Hospital acquired infection	1 (3)	0 (0)	Undefined	0.562
Severe wasting	11 (36)	17 (18)	2.46 (0.91–6.67)	0.083
Severe underweight	15 (55)	37 (40)	1.83 (0.75–4.55)	0.210
Death	8 (26)	5 (5)	6.12 (1.61–24.22)	0.003
Severe pneumonia	8/28 (29)	19/84 (23)	1.37 (0.45–3.90)	0.702
Abnormal mental status	5/7 (71)	13/17 (76)	0.77 (0.08–11.19)	0.795

Figures represent *n* (%) unless indicated otherwise.

CI: confidence interval; IQR: interquartile range; SD: standard deviation.

**Table 2:** Results of logistic regression analysis to explore the independent predictors of *Pseudomonas* bacteremia in under-five diarrheal children.

Clinical parameters	Adjusted OR	95% CI	*P*
Age (11.71 (4.0, 18.0) months)	0.99	0.96–1.01	0.312
Absent peripheral pulses	7.51	2.31–24.45	0.001
Severe wasting	1.86	0.63–5.46	0.260
Severe underweight	1.14	0.43–3.06	0.790
Severe pneumonia	1.63	0.58–4.57	0.357

**Table 3:** Antibiotic sensitivity, resistance and intermediate sensitivity of *Pseudomonas* species isolated from blood culture in under-five diarrheal children

Drugs	Sensitivity *n* = 31 (%)	Resistance *n* = 31 (%)	Intermediate sensitivity *n* = 31 (%)
Gentamicin	16 (52)	15 (48)	0 (0)
Ciprofloxacin	24 (77)	5 (16)	2 (7)
Ceftazidime	26 (84)	3 (10)	2 (7)
Imipenem	31 (100)	0 (0)	0 (0)
Netilmicin	22 (71)	8 (26)	1 (3)
Amikacin	22 (71)	7 (23)	2 (7)
Meropenem	27 (87)	2 (7)	2 (7)
